# The influence of chronotype on the body mass index of U.S. college students

**DOI:** 10.5935/1984-0063.20200130

**Published:** 2022

**Authors:** Myra Jane Bloom, Scarlet Rae Jost, Donald Paul Keating, Andrew Stuart Ian Donald Lang, Nancy Viola Mankin, Zachary William Mast, Ericka Rachel McMahan, Jonathan Abdou Merheb, Philip Paul Nelson, Joshua Chinweoke Nnaji, Enrique Francisco Valderrama

**Affiliations:** 1 Oral Roberts University, University Libraries - Tulsa - OK - United States.; 2 Oral Roberts University, Health, Leisure, & Sport Sciences - Tulsa - OK - United States.; 3 Oral Roberts University, Computing & Mathematics - Tulsa - OK - United States; 4 Oral Roberts University, Behavioral Sciences - Tulsa - OK - United States.; 5 Oral Roberts University, School of Engineering - Tulsa - OK - United States.

**Keywords:** Body Mass Index, Sleep Hygiene, Students, Universities

## Abstract

**Objectives:**

The relationship between a college student’s chronotype and body mass index (BMI) is important to understand for university decision makers who want to build healthy and inclusive academic communities. This study aimed to evaluate how a student’s chronotype influences their BMI.

**Material and Methods:**

Participants were college students from Oral Roberts University (n=384) with a mean age of 18.94 years, a mean BMI of 24.7kg/m^2^, and a mean morningness-eveningness questionnaire (MEQ) score of 47.65.

**Results:**

BMI values were significantly correlated with both chronotype (r=-.11, β=-.09, p=.03) and age (r=.12, β=.53, p=.02). The rate at which BMI increased with age depended upon the student’s chronotype (β=.81-.005 / MEQ, p=.005). The later the chronotype, the higher the rate of increase. Race had no significant influence on MEQ or BMI values except in the case of students who identified as Black and female. These students were found, on average, to have significantly higher BMI values (p<.01).

**Conclusion:**

For college students, BMI tends to increase over time and at a rate that is dependent upon chronotype. The later the chronotype, the faster the rate at which BMI increases. BMI values were found to be significantly higher for Black females. However, this result is potentially spurious, as BMI does not take into account differences in body composition between genders and race/ethnicity groups.

## INTRODUCTION

Body mass index (BMI) is a numerical value derived from a person’s weight in kilograms divided by the square of their height in meters. This formula is utilized in the U.S. for adults ages 18 and up to indicate a person’s percent body fat^1^. Depending on their BMI value, a person will be classified into one of the following categories: severely underweight (<16), underweight (17-18), optimal (19-24), overweight (25-29), obese (30-39), and severely obese (40-83). With body fat percentage being a reliable determinant for overall health and health risk, BMI has been used as a screening tool to easily determine health risks due to body fat, even though it actually measures how people carry their body weight on their frames (height), and is not a direct measure of percent body fat. However, the use of BMI has been a common and effective tool in population-based studies due to its wide acceptance in defining certain categories of body mass as related to health issues^2^.

The prevalence of obesity in the U.S. from 1999-2000 through 2017-2018 increased from 30.5% to 42.4%, with an increase of severe obesity from 4.7% to 9.2%^3^. With the rate of adults dealing with obesity increasing each year, it is not surprising to see that the prevalence of obesity among young adults ages 20-39 is 40.0%^3^. The rate of obesity was found to increase to 44.8% among middle-aged adults ages 40-59 and 42.8% among adults 60 years and older^3^.

The high rate of obesity and increased BMI scores for young adults coincides with a decrease in the amount of daily physical activity. Today, less than one in four males and one in five female university students meet recommended criteria for exercise frequency, intensity, and duration^4^. This reinforces the need for students to remain physically active during their university years in order to avoid potential serious health consequences later in life^4^. The majority of American adults (81.8%) do not get the recommended amount of daily physical activity^5^. Despite not reaching regular physical activity requirements, many Americans believe that they are acquiring a satisfactory exercise quantity^6^. However, with physical inactivity being the fourth leading cause of death worldwide, it is imperative we find solutions to address the pandemic of physical inactivity^7^.

BMI as a measure has several limitations. One limitation of particular importance to this study is the variation found in the correlation between percent body fat and BMI for people from different ethnic groups and populations. For example, Black women differ from other women in muscle mass, fat distribution, bone mineral density, and bone mass, which means that for a given BMI, Black women have significantly less percent body fat than women of other races^8-11^.

Chronotype is a measure of a person’s natural disposition with regard to their sleep-wake cycle and their preference for morning or evening activities^12^. Early chronotypes (larks) have an easier time waking up in the morning, while late chronotypes (owls) prefer to sleep in. Chronotype, typically measured on a scale from 16 to 86^12^, varies significantly by sex and age but reaches a peak in lateness at 18.4 years old for females and 19.2 years old for males^13^.

Chronotype has been found to influence sleep quality, academic performance, and mental health^14,15^ as well as playing a significant role in other lifestyle-related behaviors and outcomes, including physical activity^16^ and BMI^17-20^.

## MATERIAL AND METHODS

### Participants

The data in this study were collected from 384 university students enrolled in health and physical exercise (HPE) courses at a mid-sized university in the West South Central United States (14% international). The data are from a two-semester period (fall 2019 & spring 2020) and were collated and de-identified by members of the institutional research team before being given to the research team for analysis. This study does not include data from students who opted out (the default option), students with BMI values below 14.5kg**/** m^-2^ or above 49.4kg/m^-2^, and students whose age was below 16 or above 24. The final de-identified dataset is available as open data (CC0) via figshare^21^. The protocol of this study was approved by the University’s Institutional Review Board. The demographics of the study participants are presented in Table 1.

**Table 1:** Summary of study participant demographic data.

Male: N=150 (39.1%)		Female: N=234 (60.9%)		Total: N=384 (100%)	
Black	26 (6.8%)	Black	34 (8.9%)	Black	60 (15.7%)
Hispanic	10 (2.6%)	Hispanic	7 (1.8%)	Hispanic	17 (4.4%)
White	80 (20.8%)	White	126 (32.8%)	White	206 (53.6%)
Other	34 (8.9%)	Other	67 (17.4%)	Other	101 (26.3%)

## MEASURES

As part of the institution’s Whole Person Education program, various health and wellness indicators are assessed and discussed in required HPE activity courses. These measures include BMI and chronotype and the majority of students (over 95%) gave permission, by opting in, to use these measures as part of this study. The summary statistics of the data collected can be found in Table 2 for the following variables:

**Table 2:** Summary statistics for the data used in this study, N=384.

	Female, N=234		Male, N=150		T-test		Total, N=384	
Var.	Mean	SD	Mean	SD	t	p	Mean	SD
Age	18.84	1.24	19.11	1.37	-1.92	0.06	18.94	1.30
BMI	24.69	6.27	24.65	5.24	0.07	0.94	24.70	5.89
MEQ	46.86	7.35	48.87	7.46	-2.59	0.01*	47.65	7.45

* *p*<.05.

- **Sex:** the sex of the student was encoded as “0” for female and “1” for male. Data were collected from 234 females (61%) and 150 males (39%).
- **Age:** the age of the student, in years, approximately one week before each HPE course ended. The study participants had a median age of 19 years and a mean age 18.94 years with a standard deviation of 1.3 years.
- **BMI:** the body mass index (BMI) of study participants. BMI is taken and recorded during a lab and quiz questions test the students’ knowledge about it. The majority of instructors assist students in taking measured BMIs in these labs. The study participants had a median BMI of 23.2kg**/** m^-2^ and a mean BMI 24.7kg**/** m^-2^ with a standard deviation of 5.9kg**/** m^-2^.
- **MEQ:** the morningness-eveningness quotient of study participants - a measure of one’s preference for, and effectiveness in doing, morning or evening activities - as measured using the standard 19-item morningness-eveningness questionnaire^12^.
- **Race:** the self-identified race of study participants binned into the following categories: W = “White”, B = “Black” or “African American”, H = “Hispanic”, O = “Other” (Native American, Asian, Pacific Islander, two or more races, unknown/declined to answer), see (Table 1) above.

### Statistical analysis

Data handling, processing, and analyses were conducted using R version 4.0.2^22^. Sex differences were tested for by using a Welch two-sample t-test, Pearson’s correlation was used to analyze the association between all studied parameters, and analysis of variance (ANOVA) was used to test between competing models. The values *p*<.05 were considered statistically significant.

## RESULTS

**Figure 1:**
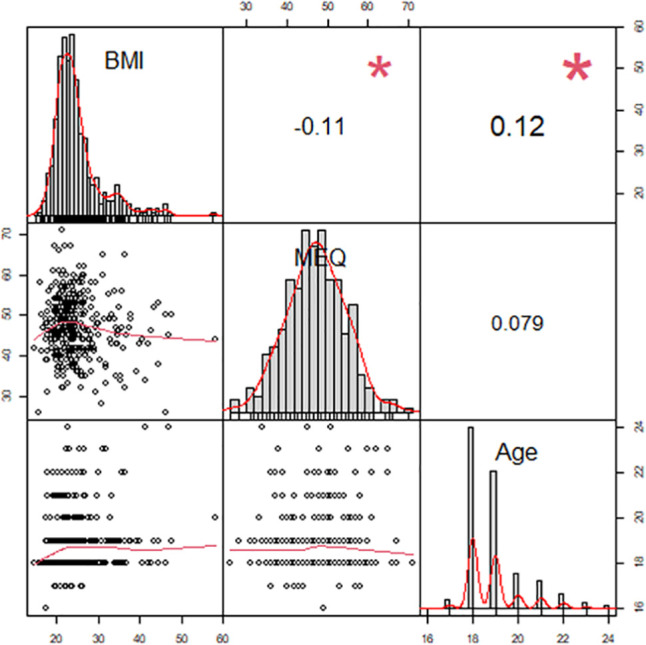
Pairwise correlation coefficients between BMI, MEQ, and age.

**Note:** *p<.05.

Out of the 384 study participants, 126 (33%) were either overweight (N=74, 19%), obese (N=40, 10%), or severely obese (N=12, 3%), and 80 (21%) were of late chronotype (MEQ<42). For the (N=384, 61% female) study participants, no significant difference was found between the sexes for body mass index values (p=.94). Males however were, on average, approximately 100 days older (p=.06) and had significantly higher MEQ scores (p=.01) than their female counterparts, see (Table 2) above. Pairwise correlation coefficients were calculated between BMI, MEQ, and age, see (Figure 1) below, with statistically significant correlations found between BMI and both MEQ (r=-.11, β=-.09, *p*=0.03) and age (r=.12, β=.53, *p*=0.02). BMI values were, on average, higher for participants who were older and had lower (later) MEQ values. The correlation between MEQ and age was not far from being considered statistically significant (r=.08, β=.45, *p*=0.12) with MEQ scores trending higher (earlier) with age.

Linear and quadratic models were created using the“ lm ” function in R for all combinations of factors available. Models where all factors were significant were noted and special attention was placed on models most relevant to the aims of our study. Linear models were analyzed and the relationship between BMI and MEQ (chronotype) was found to still be significant even when age was controlled for (*p*=0.005), see Equation 1.


(Equation 1) BMI=18.42-0.10MEQ+0.57Age

We also analyzed quadratic models, including interaction terms, and found that the rate at which BMI increased with age was higher for participants with lower (later) MEQ values (*p*=0.005), see Equation 2.


(Equation 2) BMI=13.77+(0.81-0.005MEQ) Age

**Figure 2:**
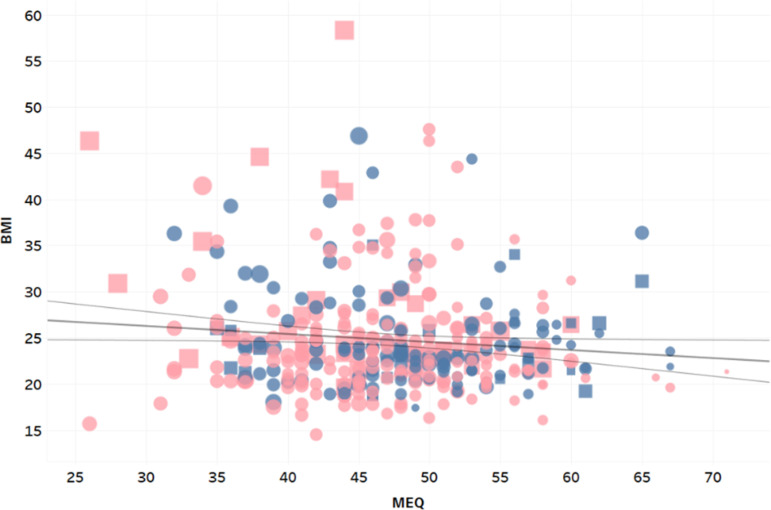
BMI vs. MEQ, points were sized by predicted BMI equation (4), colored by sex (male = blue, female = pink), and shaped by race (square = Black).

When racial differences were investigated, we found that, univariately, race had no influence on MEQ or BMI for Hispanic students (*p*=0.57; *p*=0.86). There was no significant influence of race on MEQ or BMI (*p*=0.92; β=-0.96, *p*=0.11) for those participants who identified as White. Similarly, there was not any significant difference in MEQ scores (*p*=0.91) for participants who identified as Black. However, participants who identified as Black had significantly higher than average BMI values (β=2.05, *p*=0.01). Investigating further, we found that this difference was mainly due to higher BMI values for Black females and not Black males, see Equations 3 and 4, and Figure 2. In both models (Equations 3 and 4) all variables are statistically significant (*p*<0.05) and the models’ *p*-values are *p*=0.000064 and *p*=0.000063, respectively.


(Equation 3) $$\mathrm{BMI}=16.81-0,08\;\mathrm{MEQ}+0.60\;\mathrm{Age}+\left(3.84-4.12\;\mathrm{Sex}\right)\;\mathrm{Race}.\mathrm{Black}$$

(Equation 4) $$\mathrm{BMI}=12.84+\left(0.81-0.004\;\mathrm{MEQ}\right)\mathrm{Age}+\left(3.84-4.09\;\mathrm{Sex}\right)\;\mathrm{Race}.\mathrm{Black}$$

## DISCUSSION

The study participants were all members of the institutions whole person education program, where leading a physically active and healthy lifestyle in strongly encouraged. Even so, on average, the student’s BMI values increased with age and at a rate proportional to their MEQ score. We conjecture that this is because late chronotype college students get less sleep, make poor lifestyle choices, and get less physical activity. This finding supports the growing body of evidence that maintaining good sleep hygiene is essential for university students, especially late chronotype students, at a time of life when they find it most hard to do so^14-20^.

The significant difference in BMI values found for Black females is unreliable due to recognized issues with using BMI as a measure of percent body fat for diverse populations. This finding adds to the evidence that BMI values should be used extremely carefully (and maybe not at all) when making health and wellness related decisions or setting policies that affect Black females.

## CONCLUSION

The relationships between BMI and MEQ were investigated for 384 university students aged between 16 and 24 and with BMI values between 14.5kg/m^-2^ and 49.4kg/m^-2^. BMI was found to increase significantly with both age and MEQ score with later chronotypes gaining weight faster than their early chronotype counterparts. When controlling for sex and race no significant factors were found except for Black females who had significantly higher BMI values. The results of this study has significant implications for university communities as knowing the relationship between endogenous chronotype, BMI, and lifestyle choices can inform policy decisions related to meeting an institution’s student wellbeing and inclusivity goals.
